# Atom-Precise Ligated Copper and Copper-Rich Nanoclusters with Mixed-Valent Cu(I)/Cu(0) Character: Structure–Electron Count Relationships

**DOI:** 10.3390/molecules29030605

**Published:** 2024-01-26

**Authors:** Bachir Zouchoune, Jean-Yves Saillard

**Affiliations:** 1 Unité de Recherche de Chimie de l’Environnement et Moléculaire Structurale, Université Constantine 1 (Mentouri), Constantine 25000, Algeria; bzouchoune@gmail.com; 2 Laboratoire de Chimie Appliquée et Technologie des Matériaux, Université Larbi Ben M’Hidi-Oum El Bouaghi, Oum El Bouaghi 04000, Algeria; 3 Univ Rennes, CNRS, Institut des Sciences Chimiques de Rennes-UMR 6226, 35000 Rennes, France

**Keywords:** nanoclusters, copper, nanoalloys, electron count, superatoms, supermolecules

## Abstract

Copper homometallic and copper-rich heterometallic nanoclusters with some Cu(0) character are reviewed. Their structure and stability are discussed in terms of their number of “free” electrons. In many aspects, this structural chemistry differs from that of their silver or copper homologs. Whereas the two-electron species are by far the most numerous, only one eight-electron species is known, but more electron-rich nanoclusters have also been reported. Owing to the relatively recent development of this chemistry, it is likely that more electron-rich species will be reported in the future.

## 1. Introduction

Mixed-valent group 11 nanoclusters, i.e., clusters with a partial M(0) character, are commonly constituted by a compact [M_n_]^x+^ (n > x ≥ 0) core, protected (i.e., passivated) by an outer shell composed of ligands (of which at least some are anionic, e.g., S^2−^, SR^−^, C≡CR^−^, Cl^−^, or H^−^), and possibly supplementary metal atoms, which can be considered as being in their +I oxidation state (formally M^+^) [1,2,3,4]. The presence of these M^+^ metals at the nanoclusters’ periphery is required due to the need to coordinate some of the ligands’ lone pairs, which cannot all be coordinated by the [M_n_]^x+^ core. For example, this situation occurs when thiolate ligands are present, for stability usually requires coordinating at least two among the three thiolate sulfur lone pairs. Being generally bonded to two, three, or four ligand lone pairs, the peripheral M^+^ atoms form locally stable linear 14-electron, trigonal planar 16-electron, or tetrahedral 18-electron metal centers. Their interaction with the [M_n_]^x+^ core can be mostly viewed in first approximation as being of a metallophilic nature. In most cases, the charge of such stable and isolable species, i.e., the sum of the formal charges of the cluster’s constituents (core + ligands + peripheral metals), is 0 or close to 0.

The valence electron configuration of group 11 metals in their 0 and +I oxidation states is d^10^ s^1^ and d^10^ s^0^, respectively. Since in the mixed-valent [M_n_]^x+^ core the M oxidation state is between +I and 0 (average d^10^ s^(n−x)/n^ configuration), the valence d shell is always completely filled and, therefore, the metal–metal bonding within the [M_n_]^x+^ core is only ensured by the (n − x) valence s electrons. Being atom-precise, the clusters are also electron-precise, and the value of (n − x) is imposed by stability laws, of which the closed-shell requirement is the primary one. When the compact [M_n_]^x+^ core is of approximately spherical shape, its electronic structure can be described within the superatom qualitative model [1,2,3,4,5]. This model is based on the spherical jellium approximation [5,6,7], which consists in replacing, in the Hamiltonian part describing the [M_n_]^x+^ core, the spheroidal field generated by the compact cloud of the individual nuclei’s point charges with a smoothened averaged radial (spherical) potential. The solutions of the resulting approximate Schrödinger equation are polyelectronic wave functions that can be written on the basis of one-electron functions that remind the atomic orbitals and are called superatomic orbitals. Although of spherical symmetry, as in a polyatomic atom, the jellium potential approximating the electron–nuclei interactions in a superatom is of a different nature, resulting in the absence of conditions between the *n* and *l* quantum numbers, allowing for 1P, 2P, 3P… 1D, 2D, 3D, etc., superatomic orbitals. As for atomic orbitals, there is a level ordering for superatomic orbitals, which is quite general, at least for the lowest shells (1S < 1P < 1D < 2S < 1F < 2P < 1G, etc.). The closed-shell requirement predicts stability for any superatomic configuration corresponding to the complete filling of superatomic levels, e.g., 1S^2^, 1S^2^ 1P^6^ or 1S^2^ 1P^6^ 1D^10^. It turns out that a huge proportion of isolable atom-precise nanoclusters of Ag and Au obey this shell-filling requirement, with so-called superatomic “magic” numbers of electrons (2, 8, 18, 20, 34, 40, etc.). Some, which deviate from these electron counts, can be viewed as Jahn–Teller-distorted superatoms [8]. More importantly, it has been shown that, as atoms can assemble to form molecules, several superatoms can fuse together to generate superatomic molecules (i.e., supermolecules) [9,10,11,12]. The supermolecular electron counts resemble those of simple main-group molecules, with closed-shell configurations obeying the octet rule, with triple, double, single, or no “bonds”.

Whereas the number of known Au and Ag nanoclusters today is huge, with examples having several hundreds of metal atoms (e.g., Au_333_(SR)_79_ [13] or Ag_374_(SR)_113_Br_2_Cl_2_ [14]), their Cu homologs are comparatively much scarcer. The major reason for this likely originates from the fact that the Cu(I)/Cu(0) reduction potential of Cu is substantially lower than the corresponding Ag(I)/Ag(0) and Au(I)/Au(0) potentials [15], which makes Cu(I) more difficult to reduce [16,17,18]. Another reason might be that the chemistry of atom-precise mixed-valent copper clusters is much more recent than that of its gold and even Ag relatives. Although reviews dealing with copper nanoclusters exist [19,20,21,22,23,24,25], none of them have analyzed these species from the specific point of view of electron counting. This is why we review the structurally characterized mixed-valent Cu(I)/Cu(0) nanoclusters below and analyze their structures with respect to their electron counts. Copper-rich bi- or polymetallic species are also subsequently reviewed in a similar way. Finally, we stress the general trends within this chemistry, and we propose strategy elements to help experimentalists in their syntheses of copper species with as great a Cu(0) character as possible.

## 2. Homometallic Copper Species

Before entering into the description of clusters containing a Cu_n_ core, we would like to mention the huge family of chalcogen-bridged copper nanoclusters mainly developed during the past few decades by D. Fenske and coworkers in Karlsruhe [26,27]. In most of these compounds, copper is in its +I oxidation state and surrounded by chalcogenides or, in the case of the surface metals, by ligands such as phosphines or chalcogenolates. Very few of them, such as [Cu_58_Se_16_(SePh)_24_(Ph_2_P-C≡C-PPh_2_)_6_] [28] or [Cu_26_Te_12_(PEt_2_Ph)_12_] [29], exhibit a mixed-valent character, with two “excess” electrons that would formally correspond to two Cu(0) metals. Intriguingly, their X-ray structures do not exhibit any specific structural features (e.g., Cu...Cu shortening) as compared to their regular Cu(I) relatives. Calculations performed on such species did not find any peculiarities in the MO diagrams in the HOMO–LUMO region for these species, leading to the conclusion that the “excess” electron pair is better described by a delocalized orbital of a mixed copper–chalcogen nature, which is embedded in the cluster’s valence band [24,26]. Although of mixed-valent nature, these puzzling nanoclusters do not show any copper clustering and hence will not be considered further on in this review.

The mixed-valent homometallic copper nanoclusters structurally characterized by X-ray diffraction are listed in Table 1. Their “free” electron number, determined from their published chemical composition, varies from 1 to ~179, although the reliability of the largest values is questionable (see below). The copper oxidation state averaged over all the cluster metal atoms (including the peripheral Cu(I) atoms), which is a measure of the nanocluster global electron richness, decreases with the “free” electron count and, for a given electron count, obviously increases with nuclearity. As far as we know, the smallest non-zero electron count is that of the compound [Cu_18_H_3_(S-Adm)_12_(PPh_3_)_4_Cl_2_] (Adm = adamantly) reported by Mandal and coworkers [30]. This one-electron cluster exhibits a unique core–shell structure, the metallic framework of its Cu_10_H_3_Cl_2_ core being made of a vertex-sharing Cu_6_ octahedron and a Cu_5_ square pyramid. According to DFT calculations performed by the authors, the shared vertex can be regarded as a Cu(0) center, thus holding the unique cluster electron [30]. This unprecedented paramagnetic species is better viewed as a localized mixed-valent polynuclear complex rather than a superatom. This is why it is not reported in Table 1.

**Figure 1:**
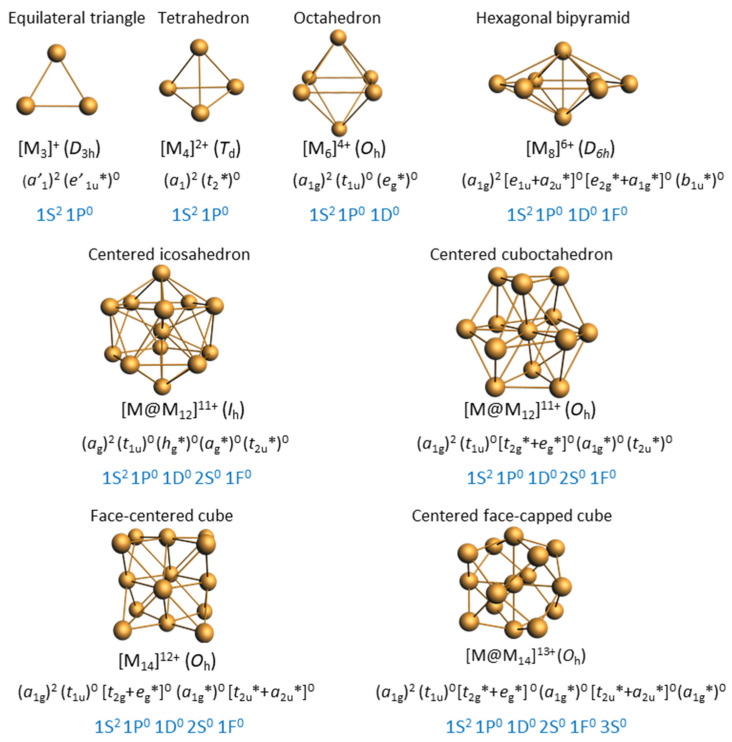
Simple [M_n_]^x+^ nanocluster cores susceptible to be stable for two electrons, with their expected (n + 1)s and corresponding superatomic electron configurations. The top line corresponds to non-centered species. Note that, although spheroidal, the hollow M_6_ and M_14_ frameworks may not properly follow the spherical jellium model. Note that the largest cores can also house eight electrons.

**Figure 2:**
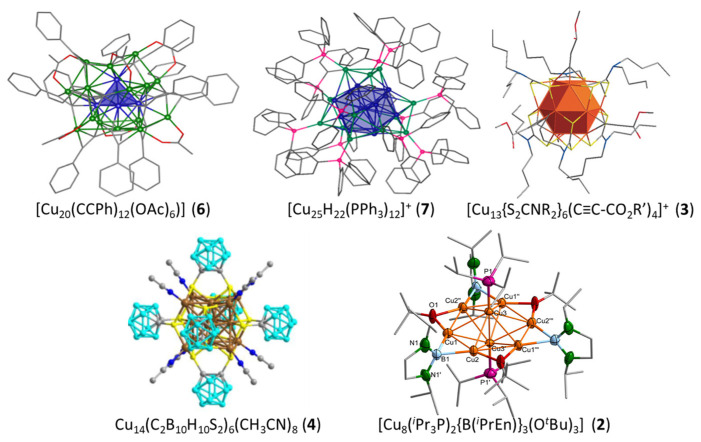
The structures of clusters **6** [31], **7 [16]**, **3 [33]**, **4 [35]**, and **2 [38]** and their tetrahedral [Cu_4_]^2+^, centered icosahedral [Cu_13_]^11+^, centered cuboctahedral [Cu_13_]^11+^, fcc [Cu_14_]^12+^, and hexagonal bipyramidal [Cu_8_]^6+^ superatomic cores, respectively. Adapted with permission from Ref. [31] (copyright 2018 American Chemical Society), from Ref. [16] (copyright 2015 American Chemical Society), and from Ref. [33] (copyright 2016 John Wiley and Sons).

The most frequent cluster electron count is two (Table 1). As illustrated in Figure 1, the smaller three-dimensional compact core able to provide a stable superatomic 1S^2^ 1P^0^ closed-shell configuration for group 11 metals is the tetrahedron. This is indeed the case of cluster **6** reported by Hayton and coworkers [31], which contains a tetrahedral [Cu_4_]^2+^ core embedded in a quite complex [Cu_16_(CCPh)_12_(OAc)_6_)]^2−^ outer shell (Figure 2). The centered icosahedral closed-packed framework is well known for housing eight electrons (1S^2^ 1P^6^ configuration) in the case of gold and silver [1,2,3,4]. However, such an architecture can also secure a large HOMO–LUMO gap for the 1S^2^ configuration ([Cu_13_]^11+^), with the two electrons housed in a strongly bonding MO of *a*_g_ symmetry (Figure 1). This situation is exemplified by clusters **7** and **8**, also made by the group of Hayton [16,32]. Figure 2 illustrates the case of **7**, whose centered icosahedral [Cu_13_]^11+^ core is protected by a [Cu_7_(CCPh)_12_(OAc)_6_)]^11−^ shell. The centered cuboctahedral framework [M_13_]^11+^ (Figure 1) is only slightly less compact than its icosahedral relative, and indeed, it is present in the clusters of type **3** (Figure 2), made by Liu and coworkers [33,34]. Interestingly, these clusters do not have any Cu(I) in their protecting outer shell, but only ligands, in contrast to **7** and **8**. The presence of dithiocarbamate ligands, which bridge the cuboctahedral square faces, might be the reason for the preference of the cuboctahedron over the icosahedron, the latter having only triangular faces. Two related two-electron clusters, **4** [35] and **5** [36], reported by Mak and coworkers, exhibit a [Cu_14_]^12+^ fcc core (Figure 1), a rather compact structure, even for such a small piece of bulk. Clusters **4** and **5** differ only in the number of terminal acetonitrile ligands on their cube vertices. The outer shell is completed by six bulky 1,2-dithiolate-o-carborane ligands which coordinate both types of metals (see the structure of **4** in Figure 2). A 14-atom fcc structure can also be viewed as an octahedron inscribed in a cube, and it is noteworthy that in their reports, the authors assume that the two cluster electrons are delocalized on the octahedron only, i.e., [Cu_6_]^2+^ (Figure 1) [35,36]. In the absence of a detailed analysis of their electronic structure, it is difficult to figure out which one of the two alternatives, [Cu_14_]^12+^ or [Cu_6_]^2+^, is the best suited for describing the superatomic core of clusters **4** and **5**.

**Figure 3:**
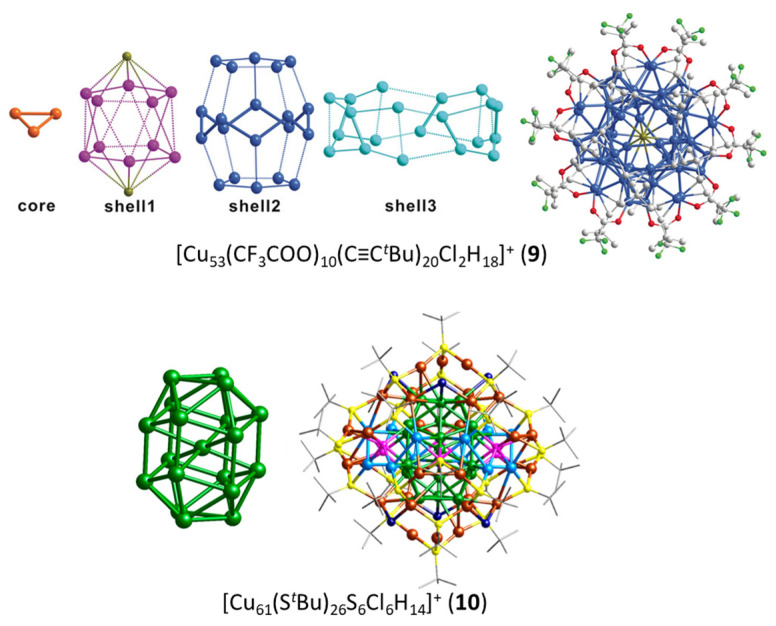
The complex anatomies of clusters **9** and **10.** Adapted with permission from Ref. [42] (copyright 2019 John Wiley and Sons) and from Ref. [43] (copyright 2019 American Chemical Society).

The four other two-electron species reported in Table 1 deviate significantly from sphericity, but one should consider that the two-electron count allows structural flexibility owing to the fact that no crucial degeneracy splitting upon symmetry lowering is expected for the 1S^2^ configuration, and sufficient compacity/connectivity is maintained. This is the case of clusters **1** and **2** reported by Kleeberg and coworkers [37,38]. The pentanuclear cluster **1 [37]** displays a distorted triangular bipyramid (Figure 1) which could be identified as a [Cu_5_]^3+^ superatomic core. However, the presence of hydride ligands (and therefore the possibility for a 0-electron core) in this compound is not to be fully excluded [37]. The octanuclear cluster **2 [38]** features a [Cu_8_]^6+^ hexagonal bipyramid (Figure 1). This oblate structural arrangement is likely to be favored by the bridging nature of the diaminoboryl and alkoxide ligands (Figure 2). The large size of the hexagonal base allows through-cage bonding between the two axial atoms, reinforcing its compact nature. Such a flat hexagonal bipyramidal structure has been reported for other noble-metal two-electron species [39,40,41]. Clusters **9** and **10** have a much larger nuclearity and a more complex structure with a large number of Cu(I) centers in their protecting shell. Cluster **9**, prepared by the group of Zheng [42], is the largest two-electron alkynyl-protected Cu nanocluster so far reported. It shows an unprecedented Cu3@Cu_10_@Cu_20_@Cu_20_ metallic core–shell1–shell2–shell3 framework (top of Figure 3). The Cu_10_ shell1 is completed with two chlorine atoms, making a Cu_10_Cl_2_ icosahedron, which is nested inside the Cu_20_ shell2 dodecahedron. The Cu…Cu core–shell2 contacts are shorter than the core–shell1 ones, making the identification of the central superatomic entity difficult. A [Cu_3_]^+^ superatomic kernel (isoelectronic to H_3_^+^) would be fine from the orbital point of view, but more complex units, such as [Cu_3_@Cu_10_]^11+^ or [Cu_3_@Cu_12_@Cu_20_]^33+^, are not to be excluded a priori. Obviously, this species deserves more attention from the point of view of its electronic structure. The two-electron copper cluster with the largest nuclearity is **10**, made by the group of Bakr [43]. It has a unique core–shell structure, with a Cu_19_ core and a giant Cu_42_(S^t^ Bu)_26_S_6_ClH_14_ outer shell (bottom of Figure 3). The Cu_19_ core describes a centered elongated triangular gyrobicupola (Cu@Cu_18_) of ideal D_3h_ symmetry, already reported, but for a non-mixed-valent Cu(I) species, in which the Cu_18_ gyrobicupola encapsulates a [Cu_2_H_5_]^3−^ unit [44]. Unfortunately, the positions of the hydrides in **10** could not be determined. Assuming that there are no hydrides inside the Cu_18_ cage, one might be tempted to assign the superatomic entity to [Cu_19_]^17+^. Alternatively, considering that the central metal in **10** is in contact with 6 among its 18 cage congeners, a [Cu@Cu_6_]^5+^ core could be also proposed. 

**Figure 4:**
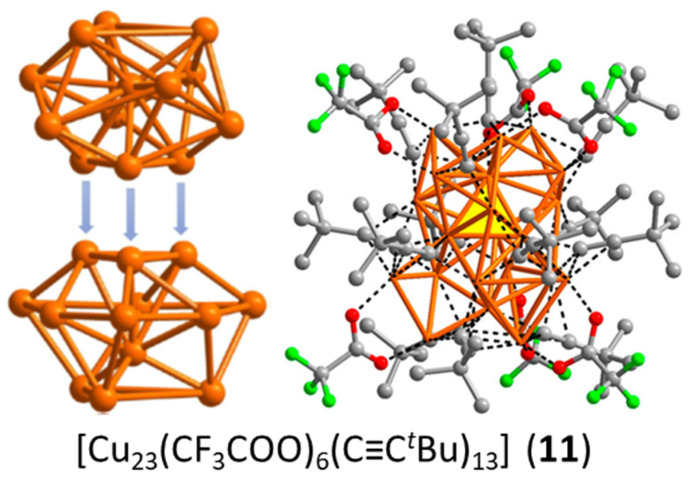
The anatomy of cluster **11**, with its face-sharing centered icosahedral and cuboctahedral units (**left**) and its yellow “central” tetrahedron (**right**). Adapted with permission from Ref. [45]. Copyright 2020 American Chemical Society.

To our knowledge, cluster **11**, made by Sun and coworkers [45], is the only four-electron copper nanocluster reported so far. Two polymorphs have been characterized in the solid state, both giving rise to the same X-ray molecular structure. The Cu_23_ framework can be described as made of a distorted centered icosahedron sharing a triangular face with a distorted centered cuboctahedron (Figure 4). Four is not a magic number for a superatom, so, at first sight, one could be tempted to see this cluster as made of the assembly of two weakly interacting two-electron spheroidal superatoms, namely a centered icosahedron and a centered cuboctahedron [9,10,11,12]. In such a case, **11** would be the supermolecular equivalent of the four-electron van der Waals He_2_ dimer. However, after a detailed analysis of the Cu…Cu distances and of their DFT-computed Cu atomic charges, the authors came to the conclusion that the four electrons are located on the Cu_4_ tetrahedron made of the center of the icosahedron and the triangular face which is shared by the two polyhedra. Yet, the existence of a tetrahedral [Cu_4_]^0^ core raises questions. Indeed, the favored electron count for such a four-orbital (4s) system is 2 ([Cu_4_]^2+^, as in compound **6**; see also Figure 1), with the two electrons occupying a strongly bonding *a’*_1_ orbital (in ideal *T*_d_ symmetry). Adding two supplementary electrons results in the partial occupation of a substantially antibonding degenerate *t*_2_* level, inducing strong Jahn–Teller distortion towards an open (butterfly) structure. Assuming additional participation of the 4p AOs of Cu does not cancel the above reasoning. So, in our opinion, a complete rationalization of the electron count of this species is still missing. 

The same group reported the eight-electron cluster **12** with 1S^2^ 1P^6^ configuration [17]. It features a centered icosahedral [Cu_13_]^5+^ core passivated by a chiral [Cu_18_(C≡CPh-OMe-4)_21_(dppe)_3_]^3−^ outer shell. The centered icosahedral [M_13_]^5+^ motif is by far the eight-electron superatomic core the most encountered in Au and Ag nanocluster chemistry [1,2,3,4], but it is highly remarkable that this very recently reported compound is the so far unique example ever reported in the case of Cu.

**Figure 5:**
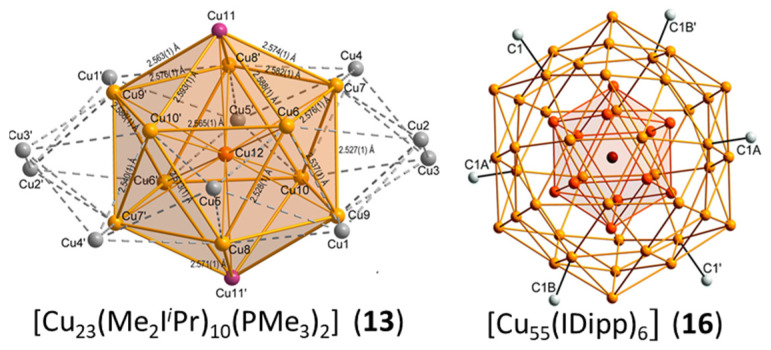
The Cu@Cu_12_@Cu_10_ and Cu@Cu_12_@Cu_42_ metal frameworks of clusters **13** and **16**, respectively. Adapted with permission from Ref. [37] (Copyright 2018 American Chemical Society) and from Ref. [46] (Copyright 2011 Royal Society of Chemistry).

Cluster **13** reported by Kleeberg et al. [37] displays a Cu_13_@Cu_10_ metal framework in which the Cu_13_ unit is a centered icosahedron. The 10 remaining Cu atoms make a crown around the icosahedral unit in such a way that each of them caps one equatorial face of the icosahedron, making a Cu_23_ framework of *D*_5d_ ideal symmetry (Figure 5, left). The two phosphines are bonded to the two icosahedral metals lying on the five-fold axis, and the ten NHC ligands are bonded to the ten crown atoms. This compound, which was obtained in low yield as a byproduct resulting from the decomposition of a copper boryl complex, is lacking characterization. In particular, the presence of hydrides on the cluster periphery is not to be excluded [37], and no indication of paramagnetism is provided, in spite of its supposed odd electron number. In a group 11 superatomic core M_n_, an electron count larger than n is not expected, owing to the spheroidal and compact nature of M_n_. Indeed, assuming that metal–metal bonding is ensured by the metal valence s AOs (and electrons), such a structural arrangement will generate a number of bonding MOs lower than (or equal to) n/2, all the other combinations being antibonding. The expected favored number of electrons for M_n_ should therefore be lower than (or equal to) n. Considering only the Cu_13_ centered icosahedron in **13** as being the superatomic core would result in a maximum of eight cluster electrons (i.e., [Cu_13_]^5+^, see Figure 1), an unrealistic value that would require the presence of 15 hydrides (or counter-anions) to balance the charges of the core and of the 10 Cu^+^ atoms. Since supplementary electrons on Cu_13_ would occupy antibonding orbitals (see Figure 1), one is therefore led to consider the Cu_10_ shell as part of the superatomic core. Assuming the Cu_13_@Cu_10_ framework is spheroidal enough, the 20-electron “magic” count (1S^2^ 1P^6^ 1D^10^ 1S^2^) appears reasonable. However, this oblate cluster might sufficiently deviate from sphericity to allow non-“magic” closed-shell electron counts that would result from Jahn–Teller splitting of degenerate (e.g., 1F) orbitals. From this point of view, a closed-shell electron number of 22 (or even 24) is not to be excluded. All these possible electron counts would of course require the presence of a certain number of hydrides (or counterions) for balancing the metal core charge. The same group also reported the large NHC-protected cluster **16** [46]. Its Cu_55_ framework, of ideal *I*_h_ symmetry, features a Cu_13_@Cu_42_ metal framework (Figure 5, right). The central Cu_13_ unit is a centered icosahedron, and the atoms of the Cu_42_ shell are capping all the icosahedron vertices (12) and edges (30). Such an M_55_ arrangement of ideal *I*_h_ symmetry is highly compact and referred to as a MacKay solid [47]. This compound was also obtained with low yield and solely characterized through X-ray diffraction analysis [46], unfortunately leaving its precise composition questionable. The closest “magic” number to 55 is 58, but the large number (36) of uncoordinated (bare) Cu atoms on the Cu_42_ shell tends to suggest that other light atoms (hydrides?) might be present in the cluster outer shell, thus reducing the cluster electron count substantially. In the same paper, the authors reported the giant NHC-protected prolate multishell cluster **17** identified as [Cu_179_(IDipp)_12_] [46]. Unfortunately, this very unique species was characterized only through a disordered X-ray structure. which must be considered with the necessary care [46] and will not be commented on further here.

**Figure 6:**
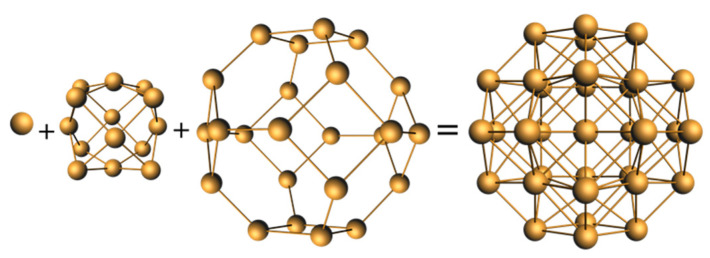
The idealized Cu@Cu_14_@Cu_24_ core of cluster **15**.

Another electron-rich cluster somehow lacking characterization is **15**, recently reported by Huang and coworkers [18]. It was described as a Cu_15_ centered hexacapped cube encapsulated in a complex shell made of two identical Cu_30_(S-Adm)_16_ semi-spherical units. A recent DFT investigation by us [48] suggests viewing the superatomic core as a compact spheroidal [Cu_15_@Cu_24_]^−^ framework, in which the Cu_24_ shell is a truncated octahedron (Figure 6). A 40-electron “magic” count would correspond to the 1S^2^ 1P^6^ 1D^10^ 1S^2^ 1F^14^ 2P^6^ configuration. However, such an electron count needs a tricationic charge to the whole cluster, which contradicts the dicationic charge suggested by the authors on the basis of mass spectrometry data [18].

**Table 1:** Structurally characterized homometallic copper clusters with some Cu(0) character. n_e_ = number of “free” cluster electrons (see text). aos = Cu average oxidation state (averaged over all the cluster metal atoms).

	Compound	n_e_	aos	Proposed Superatomic/Supermolecular Core	Ref.
**1**	[Cu_5_(Me_2_I*^i^*Pr)_3_(Bdmab)_3_]	2	0.60	Triangular bipyramid [Cu_5_]^3+^	[37]
**2**	[Cu_8_(*^i^*Pr_3_P)_2_{B(*^i^*PrEn)}_3_(O*^t^*Bu)_3_]	2	0.94	Hexagonal bipyramid [Cu_8_]^6+^	[38]
**3**	[Cu_13_{S_2_CNR_2_}_6_(C≡C-CO_2_R′)_4_]^+^ (R = *^n^*Bu, *^i^*Pr; R′ = Me, Et)	2	0.85	Centered cuboctahedron [Cu@Cu_12_]^11+^	[33,34]
**4**	Cu_14_(C_2_B_10_H_10_S_2_)_6_(CH_3_CN)_8_	2	0.86	Octahedron [Cu_6_]^4+^ or *fcc* [Cu_14_]^12+^	[35]
**5**	Cu_14_(C_2_B_10_H_10_S_2_)_6_(CH_3_CN)_6_	2	0.86	Octahedron [Cu_6_]^4+^ or *fcc* [Cu_14_]^12+^	[36]
**6**	[Cu_20_(CCPh)_12_(OAc)_6_)]	2	0.90	Tetrahedron [Cu_4_]^2+^	[31]
**7**	[Cu_25_H_22_(PPh_3_)_12_]^+^	2	0.92	Centered icosahedron [Cu@Cu_12_]^11+^	[16]
**8**	[Cu_29_Cl_4_H_22_(Ph_2_phen)_12_]^+^	2	0.93	Centered icosahedron [Cu@Cu_12_]^11+^	[32]
**9**	[Cu_53_(CF_3_COO)_10_(C≡C*^t^*Bu)_20_Cl_2_H_18_]^+^	2	0.96	Triangle [Cu_3_]^+^ or [Cu_3_ @Cu_10_]^11+^ or [Cu_3_@Cu_12_@Cu_20_]^33+^ ?	[42]
**10**	[Cu_61_(S*^t^*Bu)_26_S_6_Cl_6_H_14_]^+^	2	0.97	centered elongated triangular gyrobicupola [Cu@Cu_18_]^17+^	[43]
**11**	[Cu_23_(CF_3_COO)_6_(C≡C*^t^*Bu)_13_]	4	0.83	tetrahedron [Cu_4_]^0^ or [Cu_23_]^19+^ (see text)	[45]
**12**	[Cu_31_(C≡CPh-OMe-4)_21_(dppe)_3_]^2+^	8	0.74	Centered icosahedron [Cu@Cu_12_]^5+^	[17]
**13**	[Cu_23_(Me_2_I*^i^*Pr)_10_(PMe_3_)_2_]	23?	0?	[Cu@Cu_12_@Cu_10_]^x+^ (see text)	[37]
**14**	[Cu_53_(C≡CR)_9_(dppp)_6_Cl_3_(X)_9_] (R = Ph, C_6_H_4_Ph, X = NO_3_; R = C_6_H_4_F, X = OAc)	32	0.66	[Cu_41_]^9+^ in ABABC closed-packed stacking (see text)	[49]
**15**	[Cu_75_(S-Adm)_32_]^2+(or 3+)^	41 or 40	0.45 or 0.47	[Cu@Cu_14_@Cu_24_]^2− (or 3−)^ (see text)	[18]
**16**	[Cu_55_(IDipp)_6_]	55?	?	Icosahedral [Cu@Cu_12_@Cu_42_]^x+^	[46]
**17**	[Cu_179_(IDipp)_12_]	179?	?	See text	[46]

Abbreviations: Me_2_I*^i^*Pr = 3-bis(isopropyl)-imidazol-4,5-dimethyl-2-yliden; dmab = C_6_H_4_(NMe)_2_; *^i^*PrEn = (N*^i^*Pr)_2_C_2_H_4_; Ph_2_phen = 4,7-diphenyl-1,10-phenanthroline; Me_2_I*^i^*Pr = 3-bis(isopropyl)-imidazol-4,5-dimethyl-2-yliden; Adm = adamantyl; IDipp = C[N(C_6_H_3_-2,6-*i*Pr_2_)CH]_2_.

Finally, the 32-electron clusters **14** made by Zhang and coworkers [49] exhibit a surprising and unprecedented [Cu_41_]^9+^ core, which can be viewed as resulting from the ABABC compact stacking of hexagonal layers of 6, 7, 12, 10, and 6 atoms, respectively. At first sight, this unique framework appears not to be relevant to the superatom model. However, as mentioned by the authors, it can also be described as containing a construct made of the fusion of four centered anticuboctahedra and one centered cuboctahedron, the five polyhedra centers being arranged in a triangular bipyramidal fashion [49]. Such a description might allow viewing these species as supermolecules, the nature of which remains to be identified.

## 3. Cu-Rich Hetero-Metallic Species

### 3.1. Copper-Rich Clusters Doped with Noble Metals

The synthesis of alloys of noble metal atom-precise clusters has been tremendously developing during the last decade, owing to the opportunity it offers to tune the properties of a given system at the level of atomic precision. A huge number of mixed-valent alloys of copper with other noble metals (mainly gold and silver) have been reported. In such alloys, copper tends as far as possible to preferentially occupy the most peripheral positions (i.e., as Cu(I)), owing to its lesser ability to maximize superatomic bonding, as compared to Ag, Au, Pd, or Pt, for example [50,51,52]. We only review below the structurally characterized copper-rich species, which are listed in Table 2. As in the case of the homometallic species, the majority of these compounds, reported in Table 2, are two-electron species. 

Zhu and coworkers have reported a series of mixed heteroleptic Au/Cu clusters co-protected by phosphines [39,53,54,55]. The mono-phosphine-protected cluster **18** features a hexagonal bipyramid (Figure 1) similar to that in cluster **2** (Figure 3a) [38]. The two apices of the bipyramid are occupied by Au-PPh_3_ groups while the thiolate ligands bridge the Cu_6_ hexagon edges. The through-cage Au-Au distance is quite short (2.574 Å), and the metallic framework can be also viewed as the assembly of two perpendicular units, a linear Au-Au dimer and a Cu_6_ plane. Although it has the same nuclearity and electron count as **18**, cluster **19** adopts a different structure [53], possibly because of the presence of diphosphine ligands. This fairly unsymmetrical and chiral architecture is made of a planar Cu_4_ semi-ring inlaid with a less planar Au_4_ kernel, both components being roughly perpendicular. In the absence of a detailed analysis of its electronic structure, it is difficult to obtain a clear understanding of where the cluster’s two electrons are nested. The diphosphine-protected clusters **20** (2 electrons) and **28** (4 electrons) exhibit related complex structural features. 

**Figure 7:**
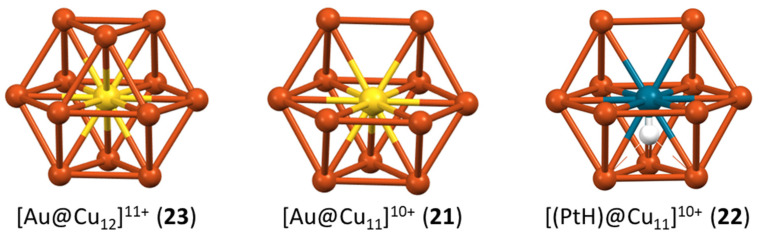
The complete and incomplete centered icosahedron in the 2-electron clusters **23**, **21**, and **22** (idealized structures).

A series of dichalcogenolate-protected monocationic clusters of type **23** have been prepared by the group of Liu [56,57,58]. They are isoelectronic and isostructural (centered cuboctahedral) to the homometallic clusters of type **3**, with the heterometal (Ag, Au) sitting at the cuboctahedron center, as expected. The neutral cluster **21**, reported by the same group [59], exhibits the same centered cuboctahedral architecture, except that one cuboctahedron vertex is not occupied (Figure 7). Thus, going from **23** to **21** consists in formally removing a cuboctahedron vertex as a Ag^+^ cation, and additionally substituting a C≡CR^−^ ligand with a chloride. The non-missing vs. missing vertex relationship between the clusters of type **23** and **21**, all having the same cluster electron count, is reminiscent of the closo vs. nido relationship in organometallic clusters obeying the Wade–Mingos rules [60]. Cluster **22** has the same structure as **21**, except that the central Au in **21** is replaced by an isoelectronic Pd-H unit in **22** (Figure 7) [61]. The encapsulated hydrogen lies close to the cuboctahedron center and should be considered as part of the superatomic core, i.e., not as an external H^−^ ligand. Its electron contributes to the cluster electron count (2 = 0 (Pd) + 1 (H) + 11 (12 × Cu) − 10 (anionic ligands)), although its 1s orbital is barely involved in the making of the superatomic orbitals [61]. The presence of encapsulated hydrides in noble metal nanoclusters is nowadays becoming not uncommon [62,63].

The clusters of type **26** are isoelectronic and isostructural to their homometallic counterpart **7** [64], with gold at the center, as expected. Cluster **25** is also a nice example of the preference for copper to occupy peripheral sites. Its 4 silver atoms form a central [Ag_4_]^2+^ tetrahedron, whereas the 15 copper atoms surround this superatomic core as Cu(I) centers [65]. A similar situation has been shown to exist in **27**, where the superatomic core has been identified as a [Ag_6_]^4+^ octahedron [66], thus leaving the four remaining Ag atoms and all the Cu atoms in the +I oxidation state. On the other hand, there seem to be two types of copper in the highly luminescent gold-centered cluster **24** [67]. Eight of them form a cube encapsulating the Au atom, with strong Au-Cu interactions, whereas the six other Cu atoms occupy more peripheral positions and are only bonded to sulfur atoms. One is thus tempted to identify the superatomic core as the centered cubic [Au@Cu_8_]^7+^ unit, although the authors, based on their XPS data, consider all the Cu atoms in the cluster to be Cu(I) [67]. Let us attempt to reconcile the two approaches in suggesting that the 1S orbital is localized on the [Au@Cu_8_]^7+^ unit, but with a large contribution of Au.

**Figure 8:**
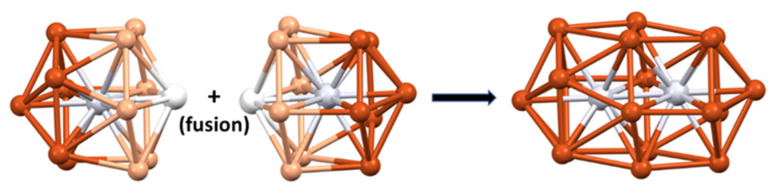
The interpenetration of two Pt-centered 13-vertex polyhedra giving rise to the [Pt_2_Cu_18_]^8+^ supermolecular core of cluster **29** through the sharing of 6 Cu and 2 Pt atoms.

The 10-electron cluster **29** [68] is a nice example of a superatomic molecule or supermolecule. Its core is made of the assembly of two interpenetrating Pt-centered 13-vertex PtCu_12_ polyhedra (Figure 8). Each individual 13-vertex polyhedron can be viewed as an icosahedron having gained one extra vertex on one of its C_5_ rings and thus can be seen as a bicapped unperfect antiprism with one pentagonal (C_5_) and one hexagonal (C_6_) face. The two centered polyhedra share their Cu_6_ hexagonal faces and two Pt centers/vertices, resulting in a [Pt_2_Cu_18_]^8+^ core, which is stabilized by a [Pt_2_Cu_16_(PET)_22_Cl_4_]^10−^ outer shell. This supermolecule is the counterpart of the 10-electron N_2_ molecule [10,12]. Its electronic structure is related to that of N_2_ in the way that the 1S and 1P orbitals of each individual superatom combine in the same way as the nitrogen 2s and 2p orbitals in N_2_. The strong interpenetration of the two polyhedra is the counterpart of the N≡N triple bond. Related 10-electron supermolecules are known [10,12]. Lower degrees of fusion between polyhedra would privilege lower closed-shell electron counts.

The 10-electron cluster **30** contains a Au_14_Cu_6_ kernel which features a tunnel-like structure composed of two Au_7_Cu_3_ units [69]. Each unit consists of a chain of three Au_3_Cu_2_ tetrahedra sharing a Au vertex. The authors described these units as six-electron systems, i.e., [Au_7_Cu_3_]^6+^ [69]. They correspond to the assembly of three non-interacting two-electron tetrahedra, the counterpart of a hypothetical He_3_ van der Waals trimer. They are protected by a passivating shell which, inter alia, contains ~1.6 Au (thus in +I oxidation state) that one have expectedto preferentially occupy core sites in the place of copper. At any rate, the majority of Cu atoms in **30** occupy Cu(I) peripheral positions.

**Figure 9:**
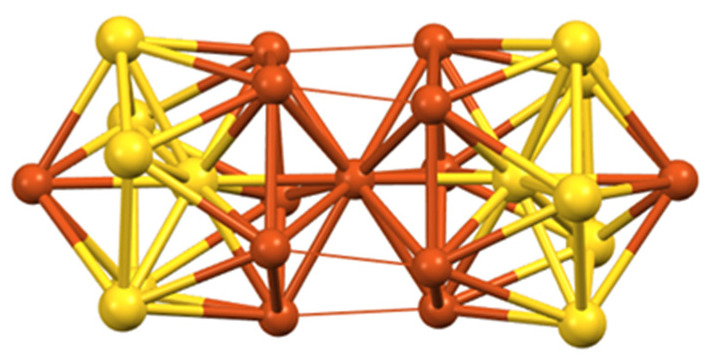
The two vertex-sharing icosahedra making the [Au_12_Cu_13_]^9+^ supermolecular core of cluster **31**.

The structure of **31** can be viewed as made of the assembly of two Au@Au_5_Cu_7_ centered icosahedra sharing a Cu vertex [70] (Figure 9). Supplementary inter-icosahedra (presumably) metallophilic contacts induced by the five bridging iodides tend to somewhat blur this picture, which is quite common in gold and silver nanocluster chemistry [4] and corresponds to two weakly interacting or non-interacting 8-electron superatoms and thus to the 16-electron [Au_12_Cu_13_]^9+^ core [10,12]. A good molecular main-group counterpart of such a supermolecule would be the 16-electron van der Waals Ne_2_ dimer. It is likely that the position of the Au atoms (Figure 9) is the most electron-rich, thus maximizing superatomic bonding within each icosahedron. 

**Figure 10:**
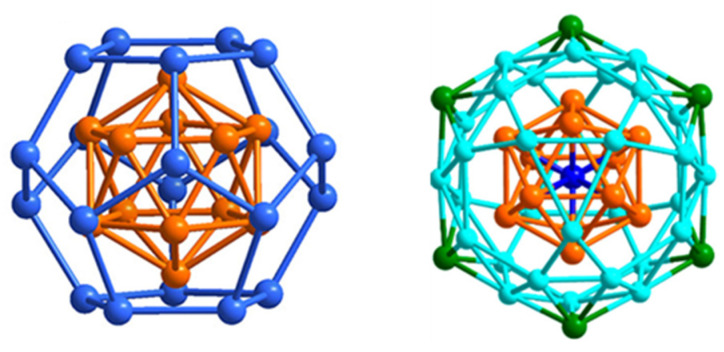
Left: The [Au@Au_12_@Cu_20_]^x+^ (x = 15–17) core of ideal *I*_h_ symmetry in clusters **32**–**34**. Right: The [Au@Au_12_@Cu_30_@Au_6_]^27+^ core of ideal *I*_h_ symmetry of clusters 35. Adapted with permissions from Ref. [71] (copyright 2017 2014 American Chemical Society) and from Ref. [75] (copyright 2017 2019 American Chemical Society).

The structures and electronic structures of clusters of types **32**, **33**, and **34** are related. The nice series of clusters 34 was made by Zheng and coworkers [71]; independently from n, they exhibit the same [Au_12_@Cu_20_] core, a gold icosahedron encapsulated in a copper pentagonal dodecahedron, in such a way that each icosahedral face is capped by a Cu atom (Figure 10, left). This construct of ideal *I*_h_ symmetry was previously observed in the hetero- and homometallic clusters [Au_12_Ag_32_(SR)_30_]^4−^ and [Au_44_(SR)_30_]^4−^ (SR=SPhF, SPhF_2_ or SPhCF_3_), for which the “magic” 18-electron 1S^2^ 1P^6^ 1D^10^ closed-shell electron configuration was ascertained by DFT [72]. This situation corresponds in **34** to a [Au_12_@Cu_20_]^14+^ superatomic core protected by 12 Cu^+^ n Au^+^ and 30 + n (SR)^−^. Whereas copper does not occupy the most inner (icosahedral) positions, in the case of n > 0, gold occupies both the inner (icosahedron) and peripheral positions. Interestingly, clusters **32**, recently isolated by Zhu and coworkers [73], have the same structure as **34** (n = 6) but have lower non-“magic” electron counts (16 and 17 for the di- and trianion, respectively). EPR experiments showed a strong signal for the 17-electron species and no signal for the 16-electron form. Even more recently, Wang and coworkers were able to crystallize the 16-electron [Au_20_._31_Cu_29_._69_(SR–O)_36_]^2−^ alloy, with a Au_12_@(Cu_17_._7_Au_22_._3_) core, showing that the dodecahedral sites in these systems are also accessible (to some extent) to gold [74]. These authors also prepared the related [Au_18_Cu_32_(SR–F)_36_]^2−/3−^. The trianion (17 electrons, type **33**), which was structurally characterized, is ESR-active, whereas the 16-electron dianion is ESR-silent. The diamagnetic behavior of the 16-electron species presented by Zhu and by Wang [73,74] indicates that their HOMO is non-degenerate. The fact that both 16 and 18 electron counts are observed for this structural composition is typical of an orbital that lies in the middle of an energy gap and can be occupied or not occupied by one or two electrons depending on the ligand nature and/or synthesis conditions. Such a hypothesis is, however, contradicted by the fact that the 1D orbitals are degenerate in *I*_h_ symmetry, not allowing one of them to be significantly destabilized above the four others unless substantial Jahn–Teller distortion occurs. 

The two clusters **35**, made by Wang and coworkers [75], show an unprecedented metal framework, Au@Au_12_@Cu_30_@Au_6_, which features a centered gold icosahedron encapsulated in a copper icosidodecahedron, the latter being surrounded by a hexameric gold ring in chair conformation (Figure 10, right). It is noteworthy that, in this case also, the most outer positions are not occupied by copper. There are 30 edges on an icosahedron, and each copper atom bridges one edge of the gold icosahedron. The external AuPh_3_ groups cap six pentagonal faces of the icosidodecahedron. This leads to the suggestion that the six external gold atoms participate in the superatomic core. The icosidodecahedron has ideal *I*_h_ symmetry, but the presence of the *S*_6_ chair-like gold ring lowers the ideal symmetry of the Au_19_Cu_30_ framework to *C*_2h_ [75]. The cluster electron count is 22, a non-“magic” number, the closest “magic” one being 20 (configuration 1S^2^ 1P^6^ 1D^10^ 2S^2^). Owing to the fact that the authors found the HOMO and LUMO to be of D and F character, respectively [75], one is tempted to suggest a Jahn–Teller distortion effect that would stabilize one of the 1F orbitals somehow below some of the occupied 1D orbitals, i.e., 1S^2^ 1P^6^ 1D^10^ 2S^2^ 1F^2^ with 1D HOMO.

**Figure 11:**
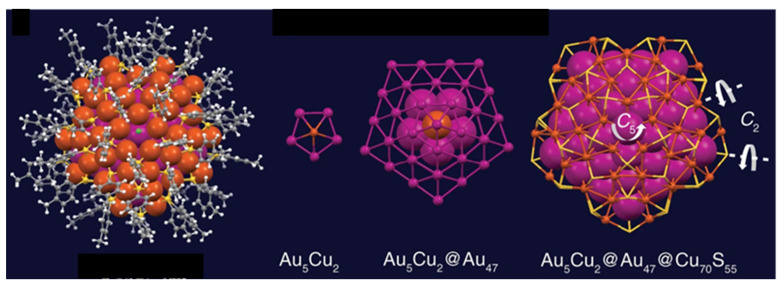
The [(Au_5_Cu_2_)@Au_47_@Cu_70_]^56+^ core of cluster **36**. Adapted with permission from Ref. [76] under Creative Commons Attribution 4.0 International License (http://creativecommons.org/licenses/by/4.0, accessed on 23 December 2023).

Cluster **36**, reported by Jin, Zhu, and coworkers, exhibits unprecedented *D*_5h_ symmetry [76]. Its multishell metal framework (Au_5_Cu_2_)@Au_47_@Cu_70_ is of ideal *D*_5h_ symmetry (Figure 11). Its inner Au_5_Cu_2_ shell is a rather compact pentagonal bipyramid, with through-cage bonding contact between the two Cu apices. This bipyramid is encapsulated within a gold shell giving rise to a two-shelled Au_52_Cu_2_ full decahedron. The presence of copper in the most inner shell is rationalized on the basis that its size is decisive in forming the perfect decahedron [76]. Cluster **36** has 68 electrons, which suggests the participation of the superatomic core of the third metal shell (Cu_70_). In addition, 68 is a “magic” number corresponding to the 1S^2^ 1P^6^ 1D^10^ 2S^2^ 1F^14^ 2P^6^ 1G^18^ 1D^10^ configuration. However, the oblate shape of this system casts doubt on an interpretation based on the assumption of sufficient sphericity.

**Table 2:** Structurally characterized mixed-valent heterometallic copper clusters alloyed with noble metals. n_e_ = number of “free” cluster electrons (see text).

		n_e_	Proposed Superatomic/Supermolecular Core	Ref.
**18**	[Au_2_Cu_6_(S-Adm)_6_(PPh_3_)_2_)]	2	Hexagonal bipyramid [Au_2_Cu_6_]^6+^	[39]
**19**	[Au_4_Cu_4_(S-Adm)_5_(dppm)_2_)]^+^	2	See text	[53]
**20**	[Au_4_Cu_6_(S-Adm)_4_(dppm)_2_Cl_3_]^+^	2	See text	[54]
**21**	[AuCu_11_{S_2_P(O*^i^*Pr)_2_}_6_(C≡CPh)_3_Cl]	2	Au-centered cuboctahedron [Au@(Cu_11_□)]^10+^ with a vacant vertex	[59]
**22**	[PdHCu_11_{S_2_P(O*^i^*Pr)_2_}_6_(C≡CPh)_4_]	2	PdH-centered cuboctahedron [(PdH)@(Cu_11_□)]^10+^ with a vacant vertex	[60]
**23**	[MCu_12_(L)_6_(C≡CPh)_4_]^+^ (M = Ag, Au, L = dtp, dtc)	2	M-centered cuboctahedron [M@Cu_12_]^11+^	[56,57,58]
**24**	[AuCu_14_(SPh*^t^*Bu)_12_(PPh(C_2_H_4_CN)_2_)_6_]^+^	2	Body-centered cube [Au@Cu_8_]	[67]
**25**	[Ag_4_Cu_15_(*R/S*PEA)_12_]^5+^	2	Tetrahedron [Ag_4_]^2+^	[65]
**26**	[AuCu_24_H_22_(L)_12_)]^+^ (L = PPh_3_, *p*-PPh_3_)	2	Centered icosahedron [Au@Cu_12_]	[64]
**27**	[Ag_10_Cu_16_(C_8_H_9_S)_16_(PPh_3_)_4_(CF_3_CO_2_)_8_]	2	Octahedron [Ag_6_]^4+^	[66]
**28**	[Au_5_Cu_6_(dppf)_2_(S-Adm)_6_]^2+^	4	See text	[55]
**29**	[Pt_2_Cu_34_(PET)_22_Cl_4_]^2−^	10	Two interpenetrated Pt-centered Cu_13_ polyhedra making a [Pt_2_Cu_18_]^8+^ dimer	[68]
**30**	Au_15_._37_Cu_16_._63_(S-Adm)_20_	12	Vertex-sharing Au_3_Cu tetrahedra making two [Au_7_Cu_3_]^4+^ “trimers”	[69]
**31**	[Au_12_Cu_13_(PPh_3_)_10_I_7_]^2+^	16	Two vertex-sharing Au-centered Au_5_Cu_7_ icosahedra making a [Au_12_Cu_13_]^9+^ dimer.	[70]
**32**	[Au_18_Cu_32_(SPhCl)_36_]^n−^ (n = 2, 3)	16–17	Au_12_ icosahedron encapsulated in Cu_20_ dodecahedron: Au_12_@Cu_20_	[73]
**33**	[Au_20_._31_Cu_29_._69_(SR–O)_36_]^2−^ [Au_18_Cu_32_(SR–O)_36_]^2−^ [Au_18_Cu_32_(SR–F)_36_]^3−^	16 17	Au-centered icosahedron Au_13_ encapsulated in a dodecahedron Cu_20_. [Au@Au_12_@Cu_20_]^16+/17+^	[74]
**34**	[Au_12+n_Cu_32_(SPhCF_3_)_30+n_]^4−^ (n = 0, 2, 4, 6)	18	Au-centered icosahedron Au_13_ encapsulated in a dodecahedron Cu_20_: [Au@Au_12_@Cu_20_]^15+^	[71]
**35**	[Au_19_Cu_30_(-C≡CR)_22_(Ph_3_P)Cl_2_]^3+^ R = SC_4_H_3_, Ph	22	Au-centered icosahedron Au_13_ icosidodecahedron Cu_30_: [Au@Au_12_@Cu_30_@Au_6_]^27+^	[75]
**36**	[Au_52_Cu_72_(SPh*^p^*Me)_55_]^+^	68	*D*_5h_-shaped (Au_5_Cu_2_)@Au_47_@Cu_70_]^56+^	[76]

Abbreviations: dtp = dithiophosphate = S_2_P(OR)_2_; dtc = dithiocarbamate = S_2_CNR_2_; *R/S*PEA = (S)-(+)-1-phenylethylamine; dppf = (diphenylphosphino)ferrocene; PET = 2-phenyl- ethanethiolate; HSPhCl = 4-chlorophenylthiophenol; SR–O = S–PhOMe; SR–F = SC_6_H_3_^3,4^F2); SPh*^p^*Me = para-methylbenzenethiolate.

### 3.2. Copper-Rich Clusters Doped with Non-Noble Metals

The group of Fischer has developed a unique chemistry of hydrocarbon-ligated intermetalloid clusters, in which two elements from groups 10–14 constitute the cluster core [77,78]. The copper-rich ones are alloys of aluminum or zinc. These compounds were prepared by reacting a (RCu)_n_ or RCuL Cu(I) species with Al_4_Cp*_4_ or Zn_2_Cp*_2_. They are listed in Table 3. The two-electron triangular cluster **37** [79] is isoelectronic and isostructural to [Zn_3_Cp*_3_]^+^ and [Zn_2_CuCp*_3_] [80], as well as to the H_3_^+^ ion, the smallest of all clusters. Such a simple arrangement (Figure 1) can be rationalized within a “spherical” 2D (planar) jellium model. On the other hand, the six-electron cluster **38** exhibits a rather unsymmetrical central framework, which can be viewed as an Al-bridged Cu_4_Al trigonal bipyramid stabilized by two Cp*, one Mes and two AlCp* ligands. It is noteworthy that in this cluster, some Al atoms belong to the six-electron core, whereas others are constituents of the peripheral ligands. The bonding within the six-electron framework can be somewhat related to that in organometallic Wade–Mingos clusters [60]. The metal core structure of **39** [81] contains at its very center a Cu_7_ unit made of two vertex-sharing Cu_4_ tetrahedra. This unit of *D*_3h_ symmetry is completed by three additional edge-bridging Cu atoms in its equatorial plane and two face-capping ZnCp* units on its *C*_3_ axis. This very peculiar structure was shown to adopt the 1S^2^ 1P_z_^2^ 2S^2^ configuration of a very elongated superatom [81].

**Figure 12:**
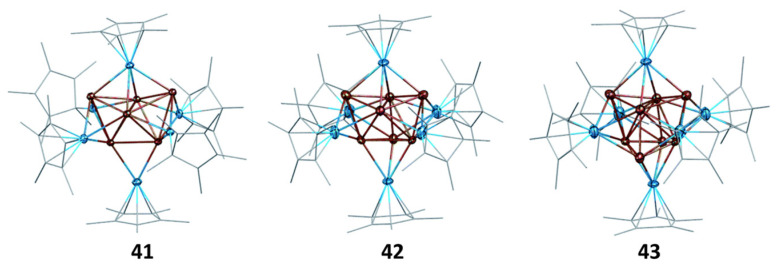
The (Cu_4_@Cu_x_)@Al_6_Cp*_6_ (x = 2, 3, 4) arrangements in the X-ray structures of **41** (X = H), **42**, and **43**, respectively. The hydride ligands in **41** and **42** were not located. Adapted with permission from Ref. [79] (Copyright 2011 Royal Society of Chemistry).

The eight-electron cluster **40** is made of a Cu_4_ tetrahedron, the faces of which are capped by four Zn atoms [82]. This Cu_4_@Zn_4_ framework of ideal *T*_d_ symmetry is perfectly suited for the 1S^2^ 1P^6^ superatomic configuration. Cluster **43** [79] features a Cu_4_@Cu_4_ tetracapped tetrahedron encapsulated within an Al_6_ octahedron (Figure 12). Its “magic” number of 20 electrons corresponds to the 1S^2^ 1P^6^ 1D^10^ 2S^2^ configuration [79]. Cluster **42 [79]** exhibits a structure similar to that of **43**, except that one of the capping Cu atoms is missing (Figure 12). DFT calculations suggest that the hydride, whose presence was ascertained by mass spectrometry and ^1^H NMR, is approximately located at the position of the missing capping copper, thus belonging to the cluster core. As in the case of cluster **22**, the hydrogen 1s electron should be considered in the cluster count, thus leading to the [(Cu_7_H)@Al_6_]^6+^ core, with the same 20-electron count as **42 [79]**. Two clusters of type **41** [83] feature a Cu_4_@Cu_2_ bicapped tetrahedron encapsulated within an Al_6_ octahedron (Figure 12). Following the analysis of **41** above, one is tempted to consider that two or three hydrides are encapsulated within the cluster core, whereas the remaining H and X ligands are regular peripheral anionic ligands. Such a situation would correspond to the 18 or 20 “magic” number of electrons, respectively. Cluster **44** adopts the same M_55_ MacKay structure [83] of ideal *I*_h_ symmetry as cluster **16**, with the Al atoms occupying 12 among the 42 positions of the metal second shell, i.e., [Cu@Cu_12_@(Cu_30_Al_12_)]^12+^ [83]. Calculations showed indeed that the Al atoms should be considered as part of the superatomic core and not as constituents of peripheral AlCp* ligands. Although the electron count of 67 is close to the 68 “magic” number, the electron configuration of **36** is fairly different from that expected for this closed-shell electron count. In fact, it approaches that of a metal particle, prefiguring the formation of a conduction band, in line with its magnetic behavior. The efficient steric protection ensured by twelve Cp* ligands annihilates any reactivity that could be anticipated for this open-shell superatomic core. This situation is unique among all the nanoclusters considered in this review.

## 4. Concluding Remarks

The structures of the copper or copper-rich nanoclusters reported above are most often different from and more complex than those encountered in the gold and silver nanocluster chemistry, making the copper nanocluster structural chemistry somewhat unique. Moreover, a large number of them are two-electron species. This is also at variance with their gold and silver counterparts, for which larger electron counts are much more frequent. The extreme scarcity of eight-electron copper superatoms [17] is particularly noteworthy, in comparison to the numerous gold and silver examples. Obviously, the lower M(I)/M(0) reduction potential of Cu (as compared to Au and Ag) renders the isolation of copper electron-rich superatoms or supermolecules more difficult [16,17,18]. When prepared by the reduction of copper salts by BH_4_^−^, the hydrides indeed behave as reducing agents (electron donors), but also quite often as coordinating ligands, including in some cases when they occupy interstitial positions [58]. This is why several of the clusters reported in Table 1 are polyhydrido species. The presence of hydrides in copper(I) clusters is also quite common [16,17,18]. Most often, the number of hydrides in a nanocluster is indirectly determined through mass spectrometry experiments, possibly completed using NMR data and, more rarely, DFT modeling. This is why, in the absence of a neutron diffraction analysis that would ascertain the hydride number and location in the cluster structure, some doubt could remain about their precise number, and consequently the number of cluster “free” electrons. For example, with two additional hydride ligands, any of the 2-electron clusters discussed above would become a Cu(I) 0-electron system. Looking for the existence of a compact metal core, the structure of which is compatible with the cluster electron count, should help remove this doubt.

In any case, electron counts much larger than two are possible, as exemplified by clusters **13–17**. Whereas **13**, **16**, and **17** are likely byproducts issued from the reductive decomposition of boryl complexes, **14** and **15** were obtained under the BH_4_^−^ reductive process. Even if the exact composition of some of these species is not ascertained (hydrides?), it is clear that clusters with an average oxidation state approaching or equal to 0 are possible. Such electron-rich species might display a spectrum of properties somewhat different from their electron-poor relatives [84]. It is likely that, owing to the strong current interest in researching the largest nanoclusters possible [85], new electron-rich mixed-valent copper nanoclusters will be reported in the future. The reduction by BH_4_^−^ using the original speed-controlling method designed by J. Huang and coworkers to isolate the electron-rich cluster **15 [18]** demonstrates that such a goal is reachable. Using other types of reducing agents and/or ligands that are different from simple thiolates (SR^−^) or alkynyls (CCR^−^) [86], such as neutral ligands (phosphines, NHCs) or dichalcogenolates (one negative charge for two chalcogen atoms) [87], might also be also a possible track. 

In the case of noble-metal heterometallic species, it is worth noting that if copper tends in general to occupy the most peripheral sites, this is not always the case, as exemplified by clusters **30**, **31**, **35**, and **37**. Such complex situations can occur in supermolecules or multishell superatoms. We must finally mention the emerging chemistry of nanoclusters made of copper alloyed with non-noble metal elements such as zinc or aluminum [77,83]. This approach has already produced a diversity of original stable frameworks with, in addition to “regular” superatomic architectures, species that are somewhat related to organometallic clusters.

An unprecedented series of 18-electron Cu_50_ nanoclusters has just been published by Sun et al. [88].

## Figures and Tables

**Table 3:** Structurally characterized mixed-valent heterometallic copper clusters alloyed with non-noble metals. n_e_ = number of cluster “free” electrons (see text).

		n_e_	Proposed Superatomic/Supermolecular Core	Ref.
**37**	[AlCu_2_Cp*_3_]	2	Triangle [AlCu_2_]^3+^	[79]
**38**	[Al_4_Cu_4_ (Mes)Cp*_5_]	6	[Cu_4_Al_2_]^4+^	[79]
**39**	[Zn_2_Cu_10_(Mes)_6_Cp*_2_]	6	*D*_3h_ [ZnCu_10_]^8+^ (see text)	[81]
**40**	[Zn_4_Cu_4_ (CN*^t^*Bu)_4_Cp*_4_]	8	Tetracapped tetrahedron [Cu_4_@Zn_4_]^4+^	[82]
**41**	[Al_6_Cu_6_H_3_XCp*_6_] (X = H, NH=CHPh)	18 or 20?	Bicapped tetrahedron@octahedron [(Cu_4_@ Cu_2_H_x_)@Al_6_]^n+^ (see text)	[83]
**42**	[Al_6_Cu_7_HCp*_6_]	20	Tricapped tetrahedron@octahedron [(Cu_4_@ Cu_3_H)@Al_6_]^6+^	[79]
**43**	[Al_6_Cu_8_Cp*_6_]	20	Tetracapped tetrahedron@octahedron [(Cu_4_@Cu_4_)@Al_6_]^6+^	[79]
**44**	[(Al_12_Cu_43_)Cp*_12_]	67	*I*_h_-shaped MacKey cluster [Cu@Cu_12_@(Cu_30_Al_12_)]^12+^	[83]

Abbreviations: Mes = mesytyl. Cp* = pentamethyl cyclopentadienyl.

## Data Availability

Not applicable.
